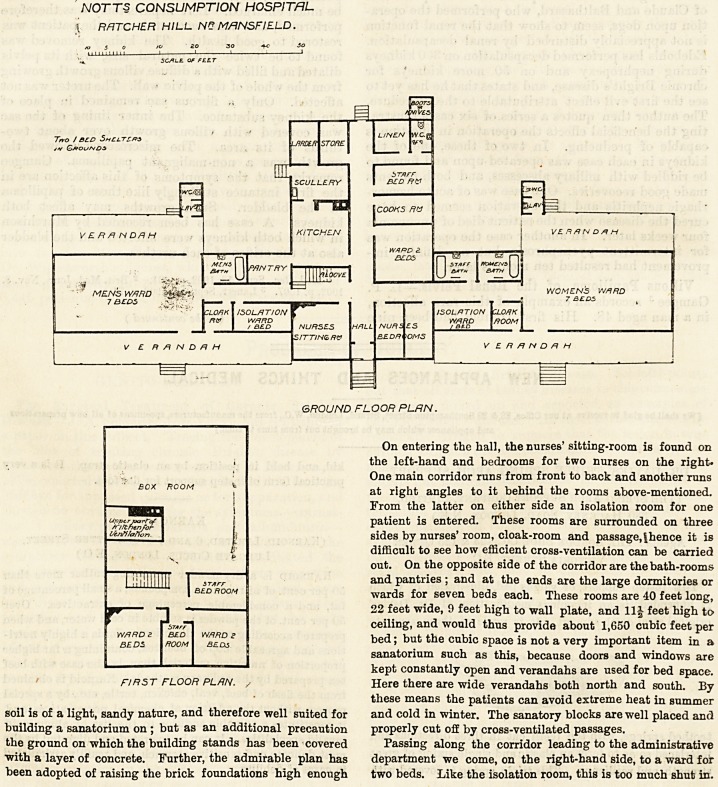# Nottinghamshire Sanatorium for Consumptives at Ratcher Hill, Mansfield

**Published:** 1903-02-14

**Authors:** 


					342 THE HOSPITAL. Feb. 14, 1903.
HOSPITAL ADMINISTRATION.
CONSTRUCTION AND ECONOMICS.
NOTTINGHAMSHIRE SAN^ORIUM FOR
CONSUMPTIVES AT RtVTCHER HILL,
MANSFIELD. V
This i3 one of the most recently constructed sanatoria in
England, and is situated on one of the highest points of
land in Nottinghamshire, being 470 feet above the sea-level.
There are 50 acres of land belonging to the institution. The
soil is of a light, sandy nature, and therefore well suited for
building a sanatorium on ; but as an additional precaution
the ground on which the building stands has been covered
with a layer of concrete. Further, the admirable plan has
been adopted of raising the brick foundations high enough
to enable workmen to walk under the floors, so that not only
is absolute dryness ensured, but the electric mains anl
water-pipes can be easily reached, if need be, without dis-
turbing any part of the structure.
The plan of the main building resembles the letter T> the
horizontal part of the letter being expanded and facing
south-east, and forming two wings, one for men and one for
women. The vertical limb of the letter runs northwards and
contains the administrative department.
On entering the hall, the nurses' sitting-room is found on
the left-hand and bedrooms for two nurses on the right.
One main corridor runs from front to back and another runs
at right angles to it behind the rooms above-mentioned.
From the latter on either side an isolation room for one
patient is entered. These rooms are surrounded on three
sides by nurses' room, cloak-room and passage,\hence it is
difficult to see how efficient cross-ventilation can be carried
out. On the opposite side of the corridor are the bath-rooms
and pantries ; and at the ends are the large dormitories or
?wards for seven beds each. These rooms are 40 feet long,
22 feet wide, 9 feet high to wall plate, and 11| feet high to
ceiling, and would thus provide about 1,650 cubic feet per
bed; but the cubic space is not a very important item in a
sanatorium such as this, because doors and windows are
kept constantly open and verandahs are used for bed space.
Here there are wide verandahs both north and south. By
these means the patients can avoid extreme heat in summer
and cold in winter. The sanatory blocks are well placed and
properly cut off by cross-ventilated passages.
Passing along the corridor leading to the administrative
department we come, on the right-hand side, to a ward for
two beds. Like the isolation room, this is too much shut in.
NOTTS CONSUMPTION HOSPITAL
\ RRTCHER HILL N& MANSFIELD.
AJ 5 O to 20 30 SO
SCALE OF FLLT
Trvo / 3CD ShClTC&S
/v Q&ou/vDS
GROUND FLOOR PLAN.
On entering the hall, the nurses' sitting-room is found on
the left-hand and bedrooms for two nurses on the right.
One main corridor runs from front to back and another runs
at right angles to it behind the rooms above-mentioned.
From the latter on either side an isolation room for one
patient is entered. These rooms are surrounded on three
sides by nurses' room, cloak-room and passage,\hence it is
difficult to see how efficient cross-ventilation can be carried
out. On the opposite side of the corridor are the bath-rooms
and pantries ; and at the ends are the large dormitories or
wards for seven beds each. These rooms are 40 feet long,
22 feet wide, 9 feet high to wall plate, and 11J feet high to
ceiling, and would thus provide about 1,650 cubic feet per
bed; but the cubic space is not a very important item in a
sanatorium such as this, because doors and windows are
kept constantly open and verandahs are used for bed space.
FIRST FLOOR PLRN. / Here there are wide verandahs both north and south. By
these means the patients can avoid extreme heat in summer
soil is of a light, sandy nature, and therefore well suited for and cold in winter. The sanatory blocks are well placed and
building a sanatorium on ; but as an additional precaution properly cut off by cross-ventilated passages.
the ground on which the building stands has been covered Passing along the corridor leading to the administrative
"with a layer of concrete. Further, the admirable plan has department we come, on the right-hand side, to a ward for
been adopted of raising the brick foundations high enough two beds. Like the isolation room, this is too much shut in.
Feb. 14, 1903. THE HOSPITAL.   343
It has, in fact, only about half of one of its sides exposed to
the open air, and such cross-ventilation as it may possess
must be into the corridors only.
The kitchen, scullery, stores, etc., are well arranged, and
are large enough for double the number of patients at
present accommodated. This is a very sensible provision, as
additions are sure to be required.
The first floor has two wards for two beds each, and also
two staff bedrooms. Apart from the sanatorium, there are
two " shelters " for one bed each. We hope it will soon be
found possible to greatly increase the number of these. At
present the total accommodation is for 24 patients.
The buildiDg is of wood. The outer covering is match-
boarded, and the inner lining is of compo-boarding over felt.
The joints of the inner skin are hidden by semi-circular
beading, and the whole is covered by a petrifying liquid.
Of course, there is a considerable air space between the two
skins, and this space could be utilised for ventilation.
The floors are covered with linoleum, and while this cover-
ing has the merits of cheapness and warmth, it would seem
from observations made at the London Hospital, that it is
well suited for a sanatorium of any kind. The intersections
of walls, floors and ceilings are properly coved.
The building is lighted by electricity, and is warmed by a
low pressure hot-water system. The water is supplied by
the Mansfield Water Works. Sewage is treated on the
bacterial system and the septic tanks are placed about two
hundred yards from the main building.
The plan of the sanatorium was sketched by the committee
and worked out by the architect of the Portable Building
Company, Fleetwood. The cost was ?220 per bed ; but it
should be taken into consideration that the administrative
department is large enough for double the number o?
patients, and that sleeping accommodation could now be
added for a much lower sum per bed. Nevertheless, after
every allowance has been made for these facts, we are dis-
posed to look upon the cost as high; and surely each patient
might at least have been provided with a separate room.
The estate on which the sanatorium stands was presented
by the Duke of Portland.

				

## Figures and Tables

**Figure f1:**